# Physiologic isolation and expansion of human mesenchymal stem/stromal cells for manufacturing of cell‐based therapy products

**DOI:** 10.1002/elsc.202100097

**Published:** 2021-10-27

**Authors:** Dominik Egger, Antonina Lavrentieva, Patrick Kugelmeier, Cornelia Kasper

**Affiliations:** ^1^ Department of Biotechnology University of Natural Resources and Life Science Vienna Austria; ^2^ Institute of Technical Chemistry Leibniz University of Hannover Hannover Germany; ^3^ Kugelmeiers Ltd. Erlenbach Switzerland

**Keywords:** 3D cell culture, expansion, hypoxia, isolation, mesenchymal stem/stromal cells

## Abstract

The utilization of mesenchymal stem/stromal cells raises new hopes in treatment of diseases and pathological conditions, while at the same time bringing immense challenges for researchers, manufacturers and physicians. It is essential to consider all steps along the in vitro fabrication of cell‐based products in order to reach efficient and reproducible treatment outcomes. Here, the optimal protocols for isolation, cultivation and differentiation of mesenchymal stem cells are required. In this review we discuss these aspects and their influence on the final cell‐based product quality. We demonstrate that physiological in vitro cell cultivation conditions play a crucial role in therapeutic functionalities of cultivated cells. We show that three‐dimensional cell culture, dynamic culture conditions and physiologically relevant in vitro oxygen concentrations during isolation and expansion make a decisive contribution towards the improvement of cell‐based products in regenerative medicine.

**Abbreviations**
GelMA: gelatin methacryloyl
HGF: hepatocyte growth factor
hPL: human platelet lysate
MSCs: mesenchymal stem/stromal cells
PGE2: prostaglandin E2
TCP: tissue culture plastic
VEGF: vascular endothelia growth factor

## 1 INTRODUCTION

Mesenchymal stem /stromal cells (MSCs) are promising candidates for a variety of therapeutic applications in the field of regenerative medicine. These non‐hematopoietic stem cells have been isolated from a variety of tissues and biological fluids such as bone marrow [1], adipose tissue [2], umbilical cord [3], dental tissues [4] and Wharton's Jelly or amniotic membrane [5] using simple procedures. MSCs have outstanding inherent therapeutically relevant properties, such as high in vitro proliferation capacities, secretion of biologically active components and differentiation potential. The traditional paradigm of replacement of damaged tissue by MSCs differentiation is currently challenged as MSCs were found to exert significant immunomodulatory effects on the innate and adaptive immune system [6], as well as multiple trophic effects stimulating regenerative actions in the neighboring cells [7]. Therefore, by July 2020 the top five medical specialties in which MSCs were used in clinical trials were traumatology, pneumology, neurology, cardiology and immunology [8]. During the COVID‐19 pandemic, MSCs were successfully used to treat one of the most severe complications of COVID‐19 which is the hyperactivation of the immune system, also known as cytokine release syndrome or cytokine storm, which can result in multiorgan failure and death [9, 10].

**FIGURE 1:**
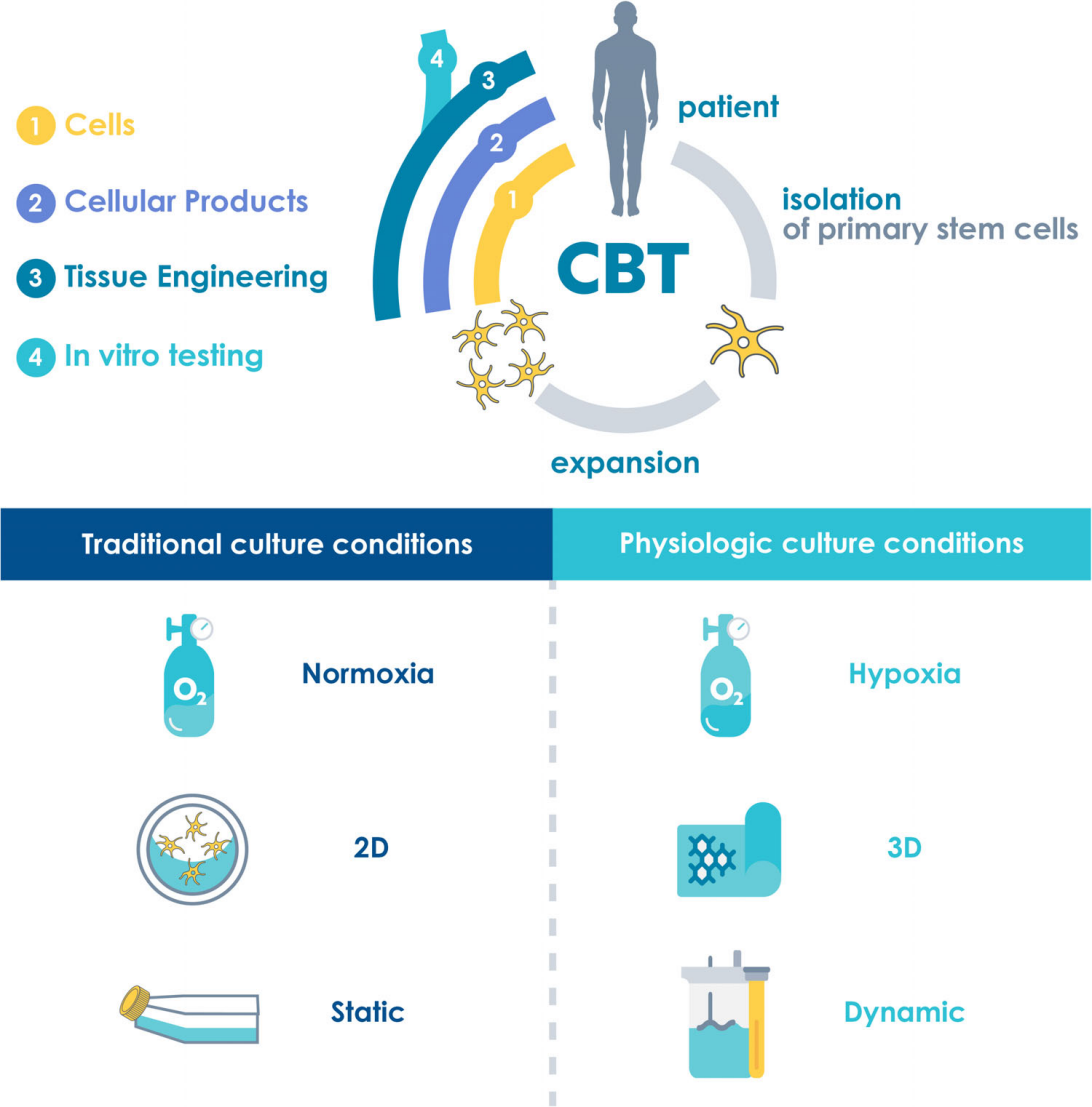
The concept of cell‐based therapies (CBT) comprises the isolation, in vitro expansion and application of stem cells. The final product can be cells, cellular products or a tissue‐engineered cell‐matrix construct for the treatment of patients or for use as in vitro models. Physiological culture conditions, such as hypoxia, 3D culture and dynamic culture, for the isolation and expansion of MSCs are essential to maintain or increase the therapeutic properties of stem cells for applications in cell‐based therapies or in vitro models

To achieve relevant cell numbers in basic research or for clinical applications, extensive *ex vivo* expansion of MSCs is required. However, polystyrene tissue culture treated plastic surfaces, such as petri dishes, well‐plates or T‐flasks, do not resemble the native environment of MSCs. Remarkably, frequent passaging on these surfaces was shown to impair therapeutically relevant stem cell properties in vitro and in vivo, induce genetic aberrations or malignant transformation of MSCs [11, 12, 13, 14]. This observation touches a critical point of the convergence of science and medicine. Stem cells with their ability to grow, differentiate and modulate hold the potential to change medicine. But the very same features can also become detrimental when clinically applied cells would turn malignant. It is thus instrumental to minimize anything that would fuel such a risk. Therefore, also culture conditions themselves must be vigorously tested whether they truly enable physiologic development. Culture conditions that reduce the risk of unintended tumorigenesis will even become legally binding as safety is the single most important factor in medicine. To increase the relevance of data from basic research and develop robust and safe large‐scale manufacturing processes, it is essential to establish physiologic culture conditions throughout the entire phase of ex vivo culture, including isolation, expansion and eventually differentiation of MSCs. Although the standard protocols for 2D isolation and expansion can be further optimized towards more physiologic culture conditions [15], true physiological conditions can only be achieved by incorporating 3D culture, dynamic culture systems, and physiologic oxygen conditions (Figure 1).

The aim of this review is to present novel concepts for the physiologic isolation and expansion of MSCs to maintain or increase therapeutically relevant functionalities. For this, we focus on the impact of 3D culture, dynamic culture and hypoxia on crucial MSC properties during in vitro expansion.

## 2 FUNCTIONAL CHARACTERISTICS OF 3D CULTURED MSCS

MSCs display enhanced therapeutically relevant functionalities when cultured in 3D culture systems [16, 17]. These effects include paracrine secretion of cytokines and chemokines [18], anti‐inflammatory [19] and immunomodulatory effects [20], reduced replicative senescence [21], differentiation potential [22], angiogenic properties [23], increased stemness [24, 25], cell survival [26] and anti‐apoptotic effects [26, 27], which have been observed in various 3D culture formats. In contrast, multiple studies prove that 2D culture leads to genetic instability and increases the risk for malignant transformation of cells [11, 12, 13, 14]. Thus, 2D culture on plastic surfaces promotes the loss of therapeutic functionalities which is disadvantageous in the context of clinical applications. Consequently, the ultimate goal is a continuous culture throughout all phases of in vitro culture, including isolation, expansion, and eventually differentiation of the cells. As the effects of 3D culture on the differentiation of MSCs have been reviewed extensively before [28, 29], this review concentrates on the effect of 3D culture during isolation and expansion.

### 2.1 3D isolation of MSCs

The isolation of MSCs from their native environment, the donor tissue, directly into a 3D environment is gaining more attention as it will allow to completely circumvent contact to 2D plastic surfaces during *ex vivo* culture. In this review we refer to these novel methods as “3D isolation” (Table 1). Egger et al. pioneered to develop and publish the first method for the isolation of MSCs from adipose tissue into a 3D hydrogel matrix [30]. For this, 5 × 5 mm pieces of adipose tissue were embedded into a hydrogel which was reconstituted from human platelet lysate (hPL) and cultured for up to 14 days. Cells migrated from the adipose tissue into to the hPL hydrogel after several days and populated the entire matrix after 14 days. The harvested cells were identified as MSCs and with this method the yield of cells was increased compared to traditional 2D explant isolation, as the cells populated the hydrogel in all three dimensions. Studies regarding the therapeutic functionalities of these 3D‐isolated MSCs are ongoing. Gharravi with colleagues constructed a perfusion bioreactor for the 3D isolation of MSCs from umbilical cord. For this, tissue pieces of 3‐4 mm were encapsulated in a 2% w/v alginate gel [31]. The cells showed enhanced proliferation and colony forming unit potential compared to static 2D explant isolation. In another study, bone marrow MSCs were isolated directly from aspirates with a known quantity of bone marrow‐derived mononucleated cells into collagen 1 scaffolds [32]. However, in this experiment the cells were directly differentiated towards the chondrogenic lineage without further analysis of the stem cell properties. Both, the 2D and 3D isolated cells, differentiated to the chondrogenic lineage while the 3D isolated cells exhibited a significantly higher aggrecan and collagen II mRNA expression. In another study, bone marrow aspirates were seeded on a porous hydroxyapatite ceramic scaffold in a perfusion bioreactor and the cells were expanded in the bioreactor while 2D expanded cells served as control [33]. Although cells in 3D during bioreactor culture did not proliferate as much as in 2D, they displayed higher clonogenicity and higher proliferation capacity after bioreactor culture. Furthermore, the authors found over 700 differentially expressed genes in 3D with a group of stemness genes being the most up‐regulated in 3D.

**TABLE 1:** Impact of 3D isolation on the functionality of MSCs

Tissue	Scaffold	Culture system	Functional effects	Ref.
Adipose tissue from abdominal plastics, 5 × 5 mm	Human platelet lysate‐based hydrogel	Static well‐plate	Isolated cells characterized as MSCs Higher yield compared to explant isolation	[30]
Umbilical cord, 3‐4 mm	Alginate hydrogel	Perfusion bioreactor	Increased proliferation and colony forming compared to static 2D explant isolation	[31]
Bone marrow aspirate with known number of mononuclear cells	Collagen 1 scaffolds	Static tissue culture flasks	Significantly increased aggrecan and collagen II mRNA expression	[32]
Bone marrow aspirate			Lower yield compared to 2D explant isolation Higher clonogenicity and proliferation after bioreactor culture Upregulation of stemness genes	[33]

Although some approaches for 3D isolation of MSCs exist, most studies did not investigate the effects of 3D isolation uncoupled from other parameters, such as dynamic conditions or inductive media components.

### 2.2 Effects of 3D expansion on therapeutic properties of MSCs

A vast number of studies report the culture of MSCs in 3D formats. However, only few studies outline the expansion of cells in 3D with a focus on therapeutic functionalities of the cells in comparison to 2D culture.

#### 2.2.1 Proliferation

Numerous studies demonstrated an increased proliferation and/or higher viability when MSCs were expanded in 3D conditions compared to 2D TCP. The substrate stiffness plays a major role as it impacts a variety of cell types. TCP has an elastic modulus of approximately 10 MPa which is rather high as most of the human tissues have an elastic modulus below 1 MPa, and only ligament, tendon and bone tissue have stiffness higher than 10 MPa [34]. MSC functionalities such as proliferation, replicative senescence and secretion of immunomodulatory cytokines were shown to be regulated by the substrate stiffness [35, 36]. For example, MSCs cultured on a novel polyacrylamide/alginate hydrogel displayed increased attachment and proliferation compared to cells cultured on 2D plastic [37]. In another study, electrosprayed genipin cross‐linked alginate–chitosan hydrogel beads were developed. MSCs displayed increased attachment to these beads and a two‐fold increase in proliferation compared to solid Cytodex 1 microcarriers while maintaining their stem cell phenotype [38]. Furthermore, MSCs embedded in fibrin hydrogels derived from different blood products demonstrated a higher proliferation compared to cells cultured on 2D TCP [39]. Although not directly tested against 2D conditions, a novel biohybrid gelatin‐methacryloyl (GelMA) hydrogel was shown to support expansion of MSCs. The synthesis and polymerization process [40] and the degree of functionalization were optimized for MSCs culture and even more positive effects were observed when the gel was formulated with human platelet lysate (hPL) [41]. The cells encapsulated in GelMA supplemented with different concentrations of hPL showed concentration‐dependent spreading, viability and proliferation. This system is especially interesting as it represents a true 3D environment were the cells are embedded and proliferate in the gel, rather than on the surface only. GelMA beads were also 3D printed layer by layer by digital light processing and investigated for the expansion of MSCs. Compared to the culture on Cytodex 1 microcarriers, the cells on the GelMA beads proliferated faster with a higher viability and higher differentiation capacity after expansion [42]. The authors consider this system as “all‐in‐one” system as the cells can also be expanded to high numbers by adding new beads while the confluently populated beads can directly be used as building blocks for differentiation and generation of larger tissues. Cell detachment using harsh enzymes such as trypsin or collagenase is known to affect the gene expression [43] and alter the surface marker profile of MSCs [44] which might affect functional characteristics of the cells. To this end, Saeed and Francini [45] presented an approach for expansion and enzyme‐free passaging of MSCs in a thermoresponsive magnetic hydrogel. The proliferation of cells in the hydrogel was approximately two‐fold higher than in 2D. Using these hydrogels, the cells could be passaged without the use of enzymes. Upon reduction of the temperature the matrix liquified, new liquid hydrogel could be added and the new diluted mix of cells and hydrogel was reseeded and polymerized by increasing the temperature back to 37°C. The cells could easily be harvested after liquefying the gel by removing the hydrogel by magnetic extraction.

#### 2.2.2 Stemness and genetic stability

Maintaining the stemness and genetic stability of MSCs during *ex vivo* culture is crucial to ensure treatment safety and maintain therapeutic stem cell functionalities. Interestingly, MSCs were shown to retain their stem cell properties longer when cultured in 3D rather than on 2D TCP. A novel approach for hydrogel‐based expansion with subsequent formation of spheroids was presented by Lee and Kim [46]. Here, MSCs were first seeded on defined shapes of a synthetic thermoresponsive hydrogel and expanded until confluence. Upon reduction of the temperature, the cell sheets detached from the hydrogel and spontaneously formed spheroids. MSCs from theses spheroids showed increased secretion of fibronectin and laminin and upregulation of stemness genes (Oct‐4, Sox‐2, and Nanog) compared to spheroids which were prepared from trypsinized single cells on ultra‐low attachment plates. Yin and Xu [47] comprehensively compared stemness properties and genetic stability during and after expansion in a polysaccharide hydrogel vs 2D plastic culture. For this, MSCs were embedded in the hydrogel or seeded on TCP and cultured for 3 weeks. Cells from the hydrogel displayed increased proliferation and viability as well as reduced replicative senescence compared to 2D plastic culture. After 3, 7, 14, and 21 days of expansion, the cells were differentiated on TCP towards adipogenic and osteogenic lineage for further 3 weeks. Interestingly, the 2D‐expanded cells exhibited a dramatic age‐dependent loss of differentiation capacity which was significantly reduced in 3D‐expanded cells. During 21 days of culture the expression of age‐related genes increased whereas the expression of stemness genes decreased in both groups. However, comparing 3D and 2D at each time point revealed that age‐related genes were downregulated and stemness genes upregulated in 3D compared to 2D after 14 and 21 days. Furthermore, the telomere length shortened faster during 2D compared to 3D expansion.

Taken together, it can be concluded that expansion in 3D systems has a beneficial impact on therapeutic functionalities of MSCs (Table 2). However, currently most systems require extensive manual handling making it difficult to use them on a routine basis or for large‐scale manufacturing of cells. To this end, hydrogels represent the most promising group of scaffolds for the 3D expansion of MSCs [48].

**TABLE 2:** Impact of 3D expansion on the functionality of MSCs

Source	Scaffold	Duration	Functional effects	Ref.
Human bone marrow	Polyacrylamide/alginate hydrogel	7 days	Increased attachment and proliferation compared cells culture on 2D plastic	[37]
Human MSCs‐hTERT cell line	Electrosprayed genipin cross‐linked alginate–chitosan microcarriers		Increased attachment and increased proliferation compared to solid Cytodex 1 microcarriers Maintaining MSC phenotype	[38]
Canine adipose tissue	Fibrin hydrogel	4 days	Increased proliferation compared to 2D static	[39]
Human adipose tissue	Gelatin methacryloyl hydrogel	7 days	Homogeneous distribution and migration of cells High viability and proliferation	[41]
Human adipose tissue	Gelatin methacryloyl hydrogel beads	7 days	Increase proliferation and higher viability Increased differentiation capacity post‐expansion	[42]
Human bone marrow	Thermoresponsive 2‐(2‐methoxyethoxy) ethyl methacrylate (MEO_2_MA) hydrogel coated with magnetic polystyrene microparticles	10 days	Increased proliferation compared to 2D static Enzyme‐free passaging	[45]
Human turbinate tissue	Thermoresponsive hydrogel composed of Tetronic‐tyramine conjugates and scaffold‐free spheroids	Twenty four hours until cell detachment and formation of spheroid, then 7 days suspension culture	Increased secretion of fibronectin and laminin Upregulation of stemness genes (Oct‐4, Sox‐2, and Nanog) compared to spheroids prepared from trypsinized single cells on ultra‐low attachment plates	[46]
Human adipose tissue from liposuctions	Commercially available polysaccharide hydrogel (TheWell Bioscience, catalog no. TWG002, Shanghai, China)	21 days	Increased proliferation and viability, and reduced replicative senescence Drastically reduced loss of differentiation capacity in cells from 3D Delayed upregulation of age‐related genes and prolonged upregulation of stemness genes in 3D Prolonged maintenance of telomer length	[47]

## 3 FUNCTIONAL CHARACTERISTICS OF BIOREACTOR‐EXPANDED MSCS

The integration of dynamic automated bioreactor systems for the manufacturing of large cell numbers is considered as a prerequisite and key technology for cell‐based therapies to enter the market [49]. Bioreactors are usually used to generate a well‐defined environment in which cells can be expanded with a high consistency and quality. In this, the environmental culture parameters play an essential role to maintain or improve therapeutic stem cell properties. Besides the standard parameters (temperature, CO_2_, O_2_, pH), other culture parameters that impact cellular functionalities are fluid shear forces, media supply, and the selection of the scaffold. The terms “microcarrier” where cells grow on a flat surface and “microsphere” where cells grow inside a hydrogel or porous material are not strictly defined nor used consistently in literature. It should be emphasized that the following paragraphs focus on dynamic approaches where cells were cultivated scaffold‐free or in and not on a 3D matrix, which excludes the use of traditional flat surface microcarriers.

### 3.1 Proliferation

Shear forces and geometrical complexity of the bioreactor bed can have adverse/antagonistic effects on cell proliferation. However, the following studies observed an increased proliferation of cells using bioreactors for the expansion of MSCs. A bi‐axial rotating bioreactor system was used for the expansion MSCs on poly (ethylene oxide terephthalate)‐co‐poly (butylene terephthalate) (PEOT/PBT). Here, an approximately 1.5‐fold increased proliferation was observed after 9 days compared to static cultivation in a well plate, while maintaining stem cell characteristics [50]. Human MSCs which were cultivated on a silk fibroin/chitosan scaffold in a spinner flask displayed an approximately 2‐, and 1.5‐fold increase in cell numbers together with a higher viability compared to static 3D and 2D well plate culture, respectively [51]. Another study demonstrated the expansion of MSCs in gelatin microbeads in a spinner flask. Here the cells, displayed a 1‐5‐fold higher proliferation index, which was derived from the number of dead cells and proliferative (EdU‐positive) cells. Also, the cells entered exponential growth 2 days earlier than the static culture, while maintaining a MSC surface marker profile, differentiation capacity and a healthy karyotype [52]. The commercially available Quantum by Terumo BCT is a hollow‐fiber bioreactor that has been used in the context of MSC manufacturing for clinical applications. Although this system represents a “pseudo‐3D” environment as the cells grow on a porous membrane rather than in a 3D matrix, it represents a perfusion bioreactor system already used for clinical applications. Here, the proliferation was twice as high as in static culture while the cells maintained their characteristic surface marker profile and differentiation capacity after extensive expansion [53].

3D aggregates of MSCs were also expanded in a suspension bioreactor or microwell plates [54]. Culture in the dynamic system resulted in a three‐fold expansion of cells compared to one‐fold in the static systems after 6 days. Also, bioreactor‐expanded cells displayed increased production of glycosaminoglycans. Thus, cultivation in 3D in bioreactors supports MSCs proliferation to obtain clinically relevant cell numbers of biologically functional cells.

### 3.2 Immunomodulatory and anti‐inflammatory effects

Shear stress which is an inherently present force in dynamic systems and it is well known that physical forces affect MSC properties, such as their differentiation capacity [55]. Furthermore, it is known that immune cells, such as B and T lymphocytes, have mechanosensing capabilities and that shear forces affect their immunomodulatory properties [56]. However, only a few studies investigated the impact of shear forces on the immunomodulatory and anti‐inflammatory properties of MSCs. In a study, where rat MSCs were cultured on a 2D polystyrene surface in a perfusion bioreactor for expansion to large scale numbers, increased secretion of prostaglandin E2 (PGE2) compared to static culture was observed. PGE2 is an anti‐inflammatory mediator which suppresses proinflammatory cytokine production of macrophages [57]. The improved potency persisted also after the mechanical stimulation was turned off.

Although studies where MSCs were cultured on 2D microcarriers in dynamic systems described increased secretion of cytokines and chemokines [58], no study investigating the effects of shear forces in a dynamic 3D culture system have been published until now, to the best of our knowledge. Thus, more research must be performed to investigate the possibilities to affect MSC immunomodulatory properties through application of defined mechanical forces.

### 3.3 Stemness

In the context of clinical applications, it is important that MSCs maintain their stemness during in vitro expansion. It was observed that MSCs lose clonogenicity, undergo replicative senescence and tend to differentiate spontaneously during 2D static cultivation [25, 26]. MSCs cultured in bioreactors were shown to maintain their stemness although they were expanded to large numbers. In a study by Zhang and Liu [59] MSCs cultured as spheroids in a microgravity bioreactor displayed enhanced proliferation, increased stemness genes (Oct‐4, Nanog, Sox‐2, Rex‐1) and multilineage differentiation potential after expansion. Furthermore, MSCs were cultured as spheroids on ultra‐low attachment plates on a rocking platform. Although this setup cannot be considered a bioreactor, it introduced convection and fluid forces to the cells. Here, the stemness genes Nanog, Oct‐4 and Sox‐2 were upregulated after culture on the rocking device. Notably, also the expression of vascular endothelia growth factor (VEGF) and hepatocyte growth factor (HGF) was elevated, indicating increased angiogenic properties of these cells [60]. However, a plate on a shaker cannot be considered a full bioreactor system.

Although in most of these studies the assessment on the impact of dynamic culture conditions were not decoupled from the impact of 3D culture or media supply, the presented studies encourage to use dynamic culture for the expansion of MSCs as therapeutic properties were often found to be elevated in MSCs during or after culture in the bioreactor systems (Table 3).

**TABLE 3:** Impact of dynamic culture on the functionality of MSCs

Source	Scaffold	Culture system	Duration	Functional effects	Ref.
Human fetal and adult bone marrow‐derived MSC	Poly (ethylene oxide terephthalate)‐co‐poly (butylene terephthalate	Bi‐axial rotating bioreactor	9 days	1.5‐fold increased proliferation compared to static cultivation in a in a well plate Maintained stem cell characteristics	[50]
Human umbilical cord blood	Silk fibroin/chitosan‐chondroitin sulfate	Spinner flask	14 days	Increased proliferation and viability compared to 3D and 2D static culture	[51]
Human Wharton's jelly	Gelatin mircobeads	Spinner flask	10 days	Increased proliferation and faster in entering exponential growth compared to 2D static Maintained MSC phenotype, differentiation capacity and healthy karyotype	[52]
Human adipose tissue from lipoaspirates	Hollow‐fiber	Quantum hollow‐fiber bioreactor	n.n.	Twice as high proliferation compared to static Maintained MSC phenotype and differentiation capacity	[53]
Human synovial fluid	Scaffold‐free spheroids	Stirred tank bioreactor	6 days	Three‐fold higher proliferation compared to static 3D Increased production of glycosaminoglycans	[54]
Rat bone marrow	Polystyrene	Perfusion bioreactor	18 h	Increases PGE2 secretion compared to static	[57]
Human adipose tissue	Scaffold‐free spheroids	Microgravity bioreactor	5 days	Increased proliferation Upregulation of stemness genes (Oct‐4, Nanog, Sox‐2, Rex‐1) Increased multilineage differentiation potential after bioreactor cultivation compared to 2D static	[59]
Human bone marrow	Scaffold‐free spheroids	Ultra‐low attachment plates on a rocking device	3 days	Upregulation of stemness genes (Nanog, Oct‐4 and Sox‐2). Increased expression of VEGF and HGF	[60]

## 4 INFLUENCE AND IMPORTANCE OF HYPOXIC CULTIVATION CONDITIONS

Another essential microenvironmental factor influencing cell physiology and behavior is in situ oxygen concentration during cultivation. Besides factors such as the use of dynamic cultivation and expansion in 3D systems, the implementation of physiological oxygen concentrations can significantly increase the therapeutic potential of MSCs and the resulting treatment outcome [61]. Although oxygen is an essential molecule for animal cells, most cells in the human organism grow and function under much lower than ambient (21%) oxygen concentrations. Depending on the tissue type, metabolic activity of surrounding cells and degree of vascularization, oxygen concentrations range from 1% (e.g. in cartilage) to the alveolar oxygen tension of 15% [62].

Moreover, in the case of pathological conditions such as wounds, tumors and infections, the oxygen level drops to critically low concentrations (below 1% or 8 mmHg PO_2_) [63, 64, 65]. During early embryonic development, before maturation and establishment of connections to maternal circulation, embryonic stem cells experience very low oxygen concentrations [65, 66]. Later, the embryo establishes connections to the maternal circulation as well as own blood vessels, and more oxygen can be delivered to the fetus. The growing cell mass, however, quickly produces hypoxic zones, which act as signaling cues for cell differentiation and proliferation of cells [67]. Here, oxygen levels function as a developmental morphogen [65, 67]. In embryogenetic angiogenesis, hypoxia is triggering migration of quiescent endothelial cells for blood vessel formation [64, 68]. Furthermore, oxygen tensions regulate limb morphogenesis ‐ here hypoxic zones act as biological signals during endochondral bone development [69, 70]. The key regulators of cell sensing and reaction on different oxygen tensions are hypoxia‐inducible transcription factors (HIFs) [67]. HIF‐1α is ubiquitously expressed and involved in the regulation of over 300 target genes [71]. Moreover, some of the target genes are transcription factors (e.g. SOX9), regulating other hundreds of genes [67, 72]. Thus, cultivation of MSCs under appropriate physiological oxygen concentrations is a logic strategy to increase their functionality.

Indeed, a growing body of research confirms the positive effects of hypoxic cultivations on MSCs isolated from different tissues [73, 74, 75, 76]. It was demonstrated that expansion in low oxygen concentrations or priming with hypoxia increases the expression of growth factors [77], pro‐angiogenic and anti‐inflammatory factors [78], the biological activity of extracellular vesicles [76], proliferation rates [73, 79] and self‐renewal capacity [80] of MSCs from different sources. Using adipose tissue‐derived MSCs which were modified with genetically encoded hypoxia sensors, it was demonstrated that HIF‐1a is stabilized in these cells starting from 7.5% O_2_ in incubator overlay [81]. Thus, cultivation in oxygen concentrations below this level has biological effects on these cells.

It is important to note that seeded cell density can also play a significant role when cells are cultivated under low oxygen concentrations in 2D cultures. In this case, local oxygen concentrations can drop further via increased metabolic consumption when high cell numbers are used. While conventional 2D cell cultures allow simpler in situ oxygen concentration control, the cultivation of cells in 3D under hypoxic conditions represents an additional challenge. During 3D cultivation, oxygen must not only diffuse from the air to the cell culture media and further to the bottom of the cell culture vessel, but oxygen molecules encounter additional diffusion barriers inside the 3D construct. Cells located in the outer layers can consume a great part of the oxygen and an anoxic core can appear if the construct size is too large. On the other hand, desired hypoxic conditions can also be achieved in 3D cell cultures without the additional regulation of oxygen concentration in the incubator. It was demonstrated that when MSCs were cultivated in cell aggregates (spheroids), hypoxia could be detected at a spheroid size of 700 μm (3 × 10^4^ cells per spheroid) in low attachment plates and at spheroid size of 800 μm (7.5 × 10^4^ cells per spheroid) in hanging drops [82]. In GelMA hydrogel constructs with the volume of 50 μL, hypoxic response was obtained by cell densities of 8 × 10^6^ cells per ml of hydrogel [81]. To summarize, the use of specific cell densities and construct sizes in 3D cell cultures, can lead to controlled hypoxia, which in addition to 3D cultivation conditions, can contribute to the manufacturing of cell‐based products of higher biological activity.

## 5 CONCLUDING REMARKS

**FIGURE 2:**
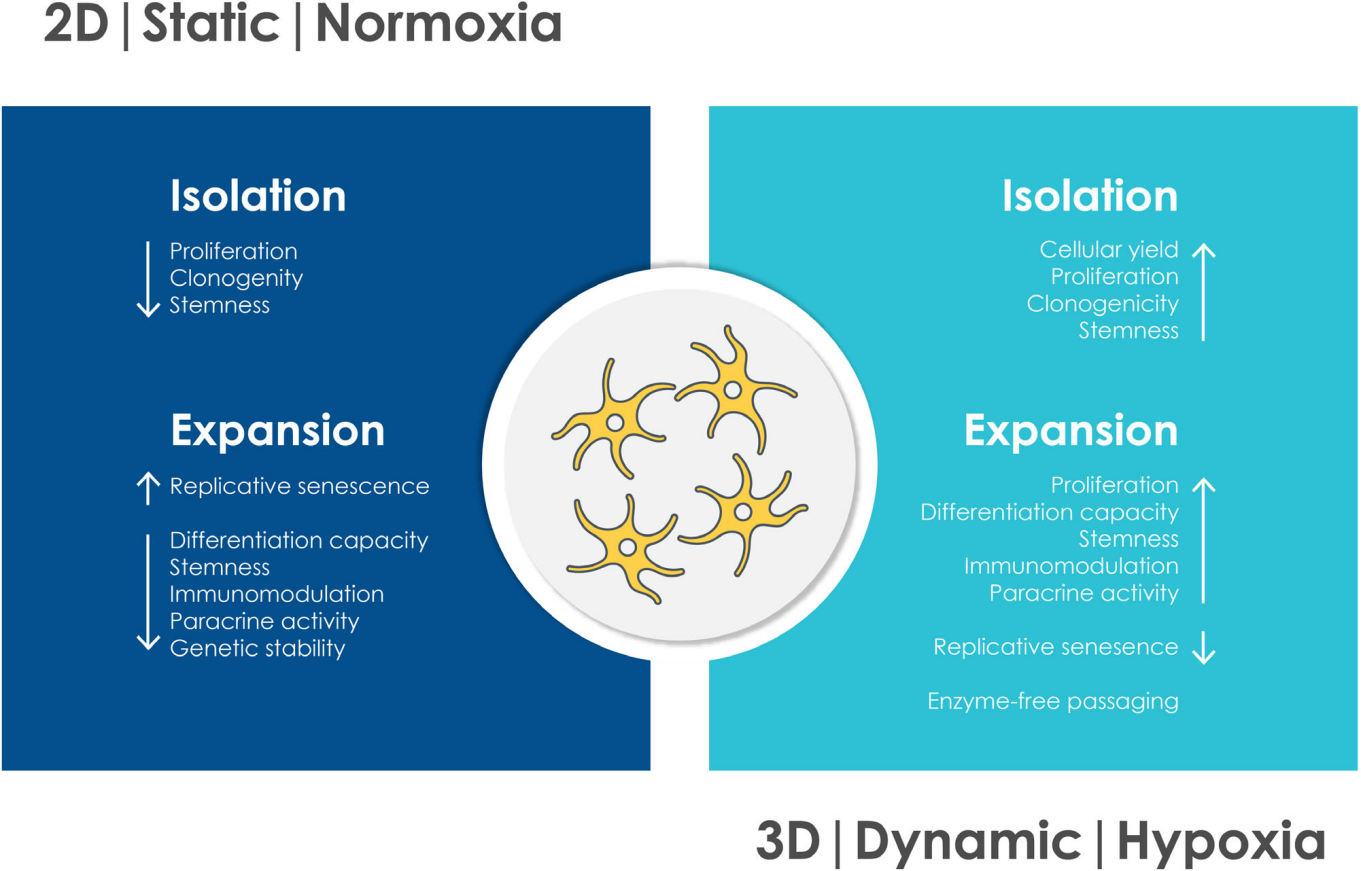
Traditional cell culture conditions, such as 2D culture on plastic surfaces, static culture and normoxia cause loss of critical therapeutic stem cell functionalities. By contrast, using 3D culture systems, dynamic culture and hypoxia can substantially increase functionality, safety and efficacy of MSCs in cell‐based therapies

Taken together, 3D culture, dynamic conditions and physiologic oxygen levels maintain and even increase therapeutically relevant properties of MSCs. By contrast, traditional culture conditions (2D plastic, static, normoxia) cause a loss of critical functionalities in MSCs (Figure 2). However, a large‐scale process for the manufacturing of healthy and potent cells and cellular products under continuous physiologic conditions is still missing. In the future, more efforts are necessary to combine the advances in 3D culture, especially in the field of hydrogels, monitoring platforms and bioreactor technology to generate a well‐defined, physiologic environment for the isolation and expansion of MSCs. Furthermore, the abundant evidence that moderate hypoxic conditions are supporting the maintenance of stem cell properties together with increased proliferation suggests the need of implementing reduced oxygen conditions during isolation and expansion. Here, novel methods to control and measure local oxygen concentrations, especially in 3D construct must be developed.

## CONFLICT OF INTEREST

Patrick Kugelmeier is Managing Partner and Director of Science at Kugelmeiers Ltd.

## AUTHOR CONTRIBUTIONS

DE, AL and CK drafted the manuscript. DE and AL performed literature search and wrote the manuscript. PK contributed to the introduction and conclusion. All authors contributed to revising the article critically and approved the final manuscript for submission.

## Data Availability

Data sharing not applicable to this article as no datasets were generated or analyzed during the current study.
